# Moral foundations underpinning attitudes toward supervised consumption services across Canada’s prairie provinces

**DOI:** 10.1186/s12954-025-01208-w

**Published:** 2025-04-29

**Authors:** Em M. Pijl, Michael Golding, Sai Krishna Gudi, Nichole Nayak, John Serieux, Christopher J. Fries, Annie Billings, Souradet Shaw, Rasheda Rabbani, Francine Laurencelle, Corey Guest, Jonny Mexico

**Affiliations:** 1 University of Manitoba, Winnipeg, Canada; 2 Winnipeg Fire Paramedic Service, Winnipeg, Canada; 3 Manitoba Harm Reduction Network, Winnipeg, Canada

**Keywords:** Harm reduction, Moral foundations, Supervised consumption services, People who use drugs, Public policy

## Abstract

**Background:**

Although there is indisputable evidence that supervised consumption services (SCS) help to keep people safe and decrease significant harms associated with substance use, the Canadian public often holds divergent and polarized views towards SCS. Polarized perspectives can be resistant to evidence and can prevent productive discourse that might otherwise lead to better public health services and outcomes.

**Objective:**

The main objective of the study was to determine the degree to which individuals' moral foundations predict attitudes toward SCS and whether attitudes are impacted by stigmatizing views of, and proximity to, people who use drugs.

**Methods:**

The study was based upon conceptual frameworks related to moral foundations theory (MFT), stigma, and personal experience with people who use drugs (PWUD), using associated instruments to determine alignment with public attitudes towards SCS. A series of hierarchical multiple linear regression analyses were employed to identify variables that significantly predict support for SCS.

**Results:**

The panel sample comprised 2116 participants from the three prairie provinces in Canada (Manitoba, n = 716; Saskatchewan, n = 700; and Alberta, n = 700). Higher scores on the *Harm/Care* and *Fairness/Reciprocity* subscales were associated with higher levels of support for SCS. Conversely, higher scores on the *Authority/Respect* and *Purity/Sanctity* subscales predicted lower levels of support for SCS. Greater support for SCS was found to be predicted by lower levels of stigma towards people who use drugs. Overall, participants from Alberta and Saskatchewan were less supportive of SCS than those from Manitoba, although Manitoba lacked an SCS at the time of the study.

**Conclusion:**

The results enhance our understanding of factors that predict support levels for SCS among the public in Canada’s Prairie Provinces. These findings can inform researchers, policy and decision-makers in developing strategies for bringing the public on board to increase the acceptance of SCS in their communities by specifically addressing underlying concerns that may not be overtly articulated by those with opposing views.

## Introduction

Overdose deaths in Canada continue to climb, with the opioid crisis claiming the lives of 4,395 Canadians in 2020, i.e.,12 opioid-related deaths every day, making the opioid crisis a leading public health concern (Canadian Centre on Substance Abuse and Addiction, 2021). The public health value of supervised consumption services (SCS) for people who use drugs has been clearly demonstrated [3, 25, 26, 36]. However, despite the considerable and growing body of evidence that these services save lives, a notable portion of the Canadian public is either ambivalent or opposed to SCS [40]. Some cities that have established SCS remain deeply divided over the local implementation of these services [6, 15, 24, 34]. Since public consultation has been a requirement by Health Canada before SCS can be established, this ongoing opposition and polarization of public discourse can, in fact, cost lives. A key strategy used by groups seeking to introduce SCS is providing expert information to gain the public’s support [43]. However, public education expounding the merits of these services often fails to overcome public opposition [32] and is not correlated to increases in public acceptance of SCS.

Controversies exist regarding the impact of SCS on the surrounding neighbouhood. SCS applications in Canada require documented consultations with neighbours within the 500-m zone around a proposed SCS, as well as a description of any intended impacts on the local area (Health [23]). The academic literature indicates there are no unintended consequences of SCS on the surrounding neighbourhood in very large population centres (Sydney Australia and Vancouver Canada) [14, 37, 45]. The grey literature and the public media in some population centres indicates there are concerns with discarded paraphernalia including needle debris, as well as a range of social disorder around SCS [4, 6, 12, 13]. As such, it is unclear whether individuals who support SCS *in theory* would support an SCS *near their home* or *near their work.*

In Canada, SCS have lower rates of public support than other harm reduction services, with only 55% of the public in favour [43]. In contrast, naloxone kit distribution has the support of 72% of Canadians, and needle distribution has 60% support. Attitudes toward SCS can be influenced by various factors, including personal values, stigma, familiarity with someone who uses drugs, media portrayal of harm reduction, and beliefs about addiction [43]. While harm reduction advocacy groups, healthcare providers and first responders support the implementation of SCS in Canadian cities, there remains a lack of consensus among the public [5, 43]. In Canada’s prairie provinces, there are currently four brick-and-mortar supervised consumption sites in Alberta and one in Saskatchewan. Until recently, Manitoba was the only province West of the Maritime provinces lacking an SCS, despite overdose deaths continuing to rise in the province [1, 8, 9]. Wild et al. [43] assessed the opinions of Canadians regarding SCS using an online panel survey, finding that prairie-dwelling respondents reported the lowest levels of support for harm reduction measures compared to all other Canadians. While stigmatizing perspectives of PWUD may be a factor in the reluctance to endorse harm reduction across the prairie provinces, there may be additional factors that contribute. Therefore, the insights generated by this research hold important implications for how public health leaders and harm reduction advocates can better engage with people who hold divergent views about SCS.

To facilitate productive discussions regarding SCS, it is vital to understand the values that underpin different perspectives, particularly in light of the fact that fact-based education (i.e. provision of factual information about the merits of SCS) does not necessarily change minds [39, 44]. By better understanding the underpinning moral motivations of those with differing views on SCS, policymakers and harm reduction advocates can have more productive dialogues with the public and address specific concerns. There is a pressing need to examine how values influence the use of scientific facts when it comes to harm reduction policy, which becomes essential when communities explore solutions to complex issues such as substance abuse.

## Methods

### Theoretical approach

The study is based upon three conceptual frameworks: Moral Foundations Theory (MFT) [18], stigmatizing view of PWUD [30, 31], and personal experience with PWUD [33]. Each framework is associated with a validated instrument. While the MFT is the primary conceptual framework of this study, we also assessed stigma and personal experiences with PWUD to see if these factors moderate perspectives originating from moral foundations.

MFT is a social psychological theory that explains the origins of and variation in human moral judgement and attitude formation. The theory postulates that moral concerns do not originate from conscious cognition but rather from the extent to which someone values and uses five psychological foundations of morality [18, 20]. MFT posits that moral judgments are primarily based on intuition rather than rational thought [20], people may have strong emotional reactions that they explain with post hoc rationalization. The five foundations are *Harm/Care, Fairness/Reciprocity, Loyalty/Ingroup, Authority/Respect, and Purity/Sanctity* [18, 20].

The *Harm/Care* foundation refers to sensitivity to signs of suffering in others. It is associated with feelings of empathy and compassion and is considered the most universally accepted moral foundation across cultures [18]. Individuals who prioritize this foundation are more likely to support harm reduction efforts, such as needle exchange [7], because of the focus on protecting individuals from harm and providing care. According to Haidt and Graham [21], compassion is not inevitable, however, and can be “turned off” by the other foundations in the theory.

The *Fairness/Reciprocity* foundation arises from humans’ history of cooperation and reciprocal altruism to [21]. This foundation is concerned with distributive justice and equity, particularly in more individualistic societies [16, 21]. This virtue can be “overridden by moral concerns from the other four systems and the many self-serving biases that lead to errors of social perception” [21], p. 104).

The *Loyalty/Ingroup* foundation arose from humans’ and other primates’ social emotions in how they live in a community with each other [18, 20]. Group cohesion and allegiance are paramount, and this foundation is closely linked to the idea of ‘ingroup’ and ‘outgroup’ distinctions [16]. People who score high on this foundation tend to be more sensitive to loyalty issues (such as nationalism or patriotism), betrayal, and intergroup conflict.

The *Authority/Respect* foundation is characterized by respecting tradition, hierarchy, and social order. It is associated with expectations of respect for authority, a sense of duty, and obedience [18]. Originating in biological realities of disease transmission [21], the *Purity/Sanctity* foundation is the concept of physical and spiritual cleanliness and is closely linked to the idea of ‘sacredness.’ People who prioritize this foundation tend to be more sensitive to issues of purity, pollution, and taboo [18], and violations of this foundation result in the emotion of disgust [21].

Graham et al. [18] assert that people’s political affiliation is associated with the weight they give to each of these foundations. For example, it is suggested that individuals who identify as politically liberal tend to place a stronger emphasis on the *Harm/Care* and *Fairness/Reciprocity* foundations and tend to be more supportive of policies that prioritize the well-being of vulnerable individuals and promote equality and social justice. On the other hand, individuals who identify as politically conservative tend to have an equal valuation of each of the foundations and place a stronger emphasis than do liberals on the *Ingroup/Loyalty*, *Authority/Respect*, and *Purity/Sanctity* foundations and tend to be more supportive of policies that promote tradition, respect for authority, and protection of moral values [18].

### Instruments

Three reliable and valid instruments were used in this study to assess moral foundations, stigma, and personal experience with PWUD. The Moral Foundations Questionnaire [MFQ30] [11, 18] is a 30-item self-report measure that assesses the extent to which individuals prioritize five moral domains: harm/care (α = 0.65), fairness/reciprocity (α = 0.61), ingroup/loyalty (α = 0.71), authority/respect (α = 0.75), and purity/sanctity (α = 0.84) [11]. The 30-item instrument consists of two 15-item subscales measuring the five moral foundations. The first subscale assesses the relevance respondents ascribe to each foundations, eliciting a response on a 7-point scale anchored by 1 = not at all relevant and 7 = extremely relevant. The stem is “When you decide whether something is right or wrong, to what extent are the following considerations relevant to your thinking?” and one sample item is, “Whether or not someone suffered emotionally.” The second subscale requires respondents to indicate on a 7-point scale the degree to which they agree or disagree with a range of moral statements. One sample item is “Respect for authority is something all children need to learn” [11, 17, 19]. In the current study, the Cronbach’s alpha values for the five moral foundation subscales were consistent with previous research (harm/care = 0.66; fairness/reciprocity = 0.63; ingroup/loyalty = 0.71; authority/respect = 0.67; purity/sanctity = 0.79).

The Perceived Stigma Towards Substance Users Scale [30, 31, 41] is an 8-item self-report measure of perceived stigma toward PWUD, with reasonable internal consistency estimates [CFI = 0.961; TLI = 0.937α = 0.80; ω = 0.80 [41]]. Individuals read 8 statements and indicate their degree of agreement or disagreement with each one, eliciting a response on a 4-point scale anchored by 1 = strongly disagree and 4 = strongly agree. A sample item is “Most people would be willing to date someone who has been treated for substance use” [30]. In the current study, the scale was found to have a Cronbach’s alpha of 0.82.

The Exposure to Drug Users Index [33] is a 7-item instrument to assess respondents’ exposure to someone who uses drugs [CFI > 0.90; IFI > 0.90; α = 0.79]. Answer choices include “Yes,” “No,” and “Not sure.” A sample item is “I have a friend who uses [substance name]” [33]. Consistent with previous research, the measure was found to have good internal consistency in current sample (α = 0.79).

### Research question and hypotheses

The main research questions of the current study were: Among the Canadian general public living on the prairies, how do people who support or oppose SCS differ in terms of moral foundations? And how might these views be moderated by their value systems, prior exposure to PWUD, and their perceived stigma towards PWUD? The hypotheses were as follows: [H_1_] People who rely on the moral foundation of *purity* are less likely to accept SCS; [H_2_] People who rely on the moral foundation of *care* are more likely to accept SCS; [H_3_] Stigmatizing attitudes have a negative impact on acceptance of SCS; [H_4_] Stigmatizing attitudes towards PWUD are correlated with the moral foundations of *care* (negative) and *purity* (positive) as well as correlated with never having ever used any substance themselves; [H_5_] Personal involvement with someone in their life who uses drugs is associated with lower stigma against people who use drugs and higher acceptance of SCS; [H_6_] People in rural communities will be less accepting of SCS than those in urban settings (i.e., rural < urban);[H_7_] People in rural communities will express moral foundations that are associated with conservative perspectives as defined by Graham et al. [18], and [H_8_] Support for a hypothetical SCS will be significantly less when the proposed SCS is near the respondent’s home or work.

### Sample

Participants were recruited through online research panels (Prairie Research Associates in Manitoba, and Leger’s Panel in Alberta and Saskatchewan) via email invitations to a random selection of potentially eligible panelists. To meet a representative sample quota of younger men, which are typically underrepresented in online surveys, a non-probability sampling approach was used and all of these eligible panelists were sent an invitation to participate. A sample of 700 adult (over 18 years of age) respondents from each of Manitoba (n = 716), Saskatchewan (n = 700) and Alberta (n = 700) was obtained. The final sample included 2116 adults, with Manitoba having an excess of 16 participants above the quota of 700 responses. Surveys were completed between September 27, 2022 and October 24, 2022. The error rate (based on a 95% confidence interval) would be ± 3.7% per province and ± 2.1% overall. The response rate with PRA was 14% and the completion rate was 79%.

To ensure that the sample accurately represented the population of each province, PRA generated a weighted dataset, with data adjusted based on the distribution of income, sex, and age according to the corresponding provincial census data. Weighting was conducted separately for each province to correct discrepancies between the sample and the census information. Furthermore, quotas were utilized to correct inherent behavioural, sociodemographic and attitudinal biases (Moser & Stuart, 1953).

A post-hoc power analysis was conducted using G*Power (version 3.1.9.4) statistical software, assessing power for different scenarios, including a multiple regression model with a medium effect size of 0.15 [7]. Given that the sample size was large enough, we used a significance level 0.01. Our post-hoc power analysis determined that we had a power of 0.99, indicating sufficient statistical power to detect a true relationship between our variables.

### Statistical analysis

Statistical analysis was performed using IBM™ SPSS (Version 27), Stata (Version 18 BE), and the programming language R (Version 4.2.3) [35]. All tests of significance were conducted at α = 0.01. All variables were summarized using descriptive statistics (mean, percentages and standard deviation) and the normality of the distributions was assessed to determine the need for parametric or non-parametric tests. A series of multiple linear regression analyses were employed to determine whether moral foundation scores were predicted by substance use, stigma, a personal history of substance use, and municipality size. These analyses included both unadjusted and adjusted models that controlled for the impact of the participant’s sex, income, and educational attainment. The results for the adjusted analyses are reported unless specified otherwise. A mixed effect logistic regression was used to compare the likelihood of supporting an SCS in general as opposed to near one’s home or work, using binarized values. Lastly, hierarchical multiple linear regression was used to identify variables that significantly predicted support for supervised consumption sites in general and to predict support for a hypothetical SCS near the participant’s home or work. In all analyses, observations were weighted by age, sex, and income to reflect the census demographics of the respondents’ province.

## Results

The final sample included 2,116 participants from Canada’s three prairie provinces (Manitoba [n = 716], Saskatchewan [n = 700] and Alberta [n = 700]). The mean age of the study participants was 48.7 years (SD = 17.7). Participants were slightly more likely to be female than male (52.4% vs. 47.6%). Most of the study participants reported their ethnicity as Western (63.9%), followed by Eastern European (16.7%). Participants were relatively evenly split across the three eligible provinces (Manitoba [33.8%], Saskatchewan [33.1%] and Alberta [33.1%], but most were living in the urban centres with populations of greater than or equal to 50,000 people (68.6%) at the time of the study. Most study participants reported having a post-secondary education (78.6%), and over half had an annual income of CAD 70,000 or more (54.7%). Detailed socio-demographic characteristics of the study participants are listed in Table 1.

**Table 1:** Descriptive statistics for the demographic variables, outcomes, and other independent variables

Variables	N = 2116	%	M (SD)
Age			48.7 years (17.7)
*Sex*			
Female	1108	52.4	
Male	1007	47.6	
Intersex	1	< 0.1	
Other	1	< 0.1	
*Ethnicity*			
Western European	1353	63.9	
Eastern European	353	16.7	
East Asian (e.g., Hong Kong, China, Japan, and Korea)	75	3.5	
First Nations, Inuit, or Métis	70	3.3	
Southeast Asian (e.g., Malaysia, Indonesia, & Philippines	42	2.0	
South Asian (e.g., India, Pakistan, Bangladesh, & Sri Lanka)	39	1.8	
*Education*			
Elementary school	9	0.4	
High School	443	20.9	
Trade, technical, or vocational certificate, apprenticeship, or pre-university degree	685	32.4	
Bachelor’s Degree	695	32.8	
Graduate Degree	283	13.4	
*Annual Income*			
$5,000–$29,999	262	12.4	
$30,000–$69,999	568	26.8	
$70,000–$99,999	381	18.0	
> $100,000	776	36.7	
*Municipality size*			
< 15,000	454	21.4	
15,000–49,999	206	9.7	
≥ 50,000	1451	68.6	
*Province*			
Alberta	700	33.1	
Saskatchewan	700	33.1	
Manitoba	716	33.8	
*Moral Foundations*			
Harm/care	2111		21.2 (4.7)
Fairness/reciprocity	2110		20.3 (4.4)
Authority/respect	2110		17.2 (5.0)
Ingroup/loyalty	2111		14.9 (5.2)
Purity/sanctity	2110		15.3 (6.4)
Stigma of addiction	2110		22.0 (3.7)
Exposure to PWUD	2098		2.5 (2.2)
*History of illicit drug use*			
Yes	389	18.4	
No	1727	81.6	
*History of cannabis use*			
Yes	1334	63.0	
No	782	37.0	
*History of prescription misuse*			
Yes	204	9.6	
No	1912	90.4	

### Predicting general support for SCS

Support for SCS, in general, was predicted by several demographic variables, which explained 8% (*p* < 0.001) of the variance as a whole. In particular, participants in Alberta (*β* = -0.18, *p* < 0.001) and Saskatchewan (*β* = −0.18, *p* < 0.01) were found to be less supportive of SCS than participants in Manitoba. Likewise, participants from both smaller (i.e., < 15,000 people; *β* = −0.10, *p* < 0.001) and medium-sized (i.e., 15,000–49,999 people; *β* = −0.08, *p* < 0.001) municipalities were less supportive of SCS compared to those from large municipalities (≥ 50,000 people).

Participants’ level of education was also found to predict general support for SCS. In general, participants with higher levels of education were more supportive of SCS than those with lower levels of education (*β* = 0.12, *p* < 0.001). Income was also significantly related to support for SCS in that individuals making between $5,000 and $29,000 annually were more supportive, on average, than individuals making $100,000 or more (*β* = 0.08, *p* = 0.003). Lastly, participant sex predicted support for SCS in the first model (i.e., block 1 in Table 2). However, it was not statistically significant after the moral foundation variables were added in the final model (i.e., block 3 in Table 2).

**Table 2:** Hierarchical regression predicting general support for supervised consumption sites

	Block 1				Block 2				Block 3			
	β	*b*	SE	*p*	β	*b*	SE	*p*	β	*b*	SE	*p*
Age	−0.04	−0.002	0.001	0.14	−0.01	−0.001	0.001	0.60	0.01	0.001	0.001	0.62
Sex	0.08	0.17	0.05	0.001	0.10	0.20	0.05	< 0.001	0.03	0.06	0.04	0.16
Education	0.12	0.10	0.05	< 0.001	0.14	0.11	0.02	< 0.001	0.06	0.05	0.02	0.003
*Province*												
Manitoba	Ref	–	–	–	Ref	–	–	–	Ref	–	–	–
Alberta	−0.18	−0.39	0.06	< 0.001	−0.16	−0.36	0.06	< 0.001	−0.08	−0.18	0.05	0.001
Saskatchewan	−0.18	−0.40	0.06	< 0.001	−0.17	−0.38	0.06	< 0.001	−0.08	−0.18	0.05	0.001
*Municipality size*												
Large municipality	Ref	–	–	–	Ref	–	–	–	Ref	–	–	–
Medium municipality	−0.08	−0.31	0.09	< 0.001	−0.08	−0.31	0.09	< 0.001	−0.04	−0.14	0.08	0.07
Small Municipality	−0.10	−0.27	0.06	< 0.001	−0.11	−0.28	0.06	< 0.001	−0.07	−0.18	0.05	0.001
*Annual Income*												
> $100,000	Ref	–	–	–	Ref	–	–	–	Ref	–	–	–
$70,000–$99,999	0.02	0.05	0.07	0.47	0.02	0.06	0.07	0.34	0.01	0.02	0.06	0.76
$30,000–$69,999	0.04	0.09	0.06	0.14	0.05	0.12	0.06	0.05	0.04	0.09	0.05	0.09
$5,000—$29,999	0.08	0.23	0.08	0.003	0.07	0.22	0.08	0.006	0.04	0.14	0.07	0.06
Stigma of addiction					−0.04	−0.01	0.01	0.10	−0.08	−0.02	0.01	< 0.001
Exposure to PWUD					0.03	0.02	0.01	0.21	−0.02	−0.01	0.01	0.44
Hx of illicit drug use					0.02	0.06	0.07	0.36	0.01	0.03	0.06	0.58
Hx of cannabis use					0.12	0.27	0.06	< 0.001	0.03	0.07	0.05	0.16
Hx of rx misuse					0.02	0.07	0.09	0.40	0.001	0.003	0.08	0.97
Harm/care									0.26	0.06	0.01	< 0.001
Fairness/reciprocity									0.17	0.04	0.01	< 0.001
Authority/respect									−0.10	−0.02	0.01	0.002
Loyalty/in-group									0.01	0.003	0.01	0.68
Purity/sanctity									–0.35	–0.06	0.005	< 0.001
R^2^ (adjusted R^2^)	0.08 (0.08)				0.11 (0.10)				0.32 (0.31)			

For the sex variable males were coded as 1 and females as 2. Large municipality= greater than 50,000 people; Medium municipality=15,000-49,999 people; Small municipality= less than 15,000 people Abbreviations: SE, standard error for the unstandardized regression coefficient (b); p,* p*-value; Ref, reference category; PWUD, people who use drugs; Hx, history; rx, prescription

The variables entered in the second block (stigma, exposure to people who use drugs, and a personal history of drug use) were found to explain an additional 3% (*p* < 0.001) of the variance in predicting participants’ support for SCS in general. Among these variables, only a history of cannabis use was found to be significantly associated with support for SCS (*β* = 0.12; *p* < 0.001). However, this relationship was not statistically significant in the third block. Surprisingly, several factors did not predict support for SCS in the second block, including stigma towards people who use drugs, greater exposure to people who use drugs, a personal history of using illicit drugs, and misusing prescription medications. However, following the addition of the moral foundation variables on the third block, more significant support for SCS was found to be predicted by less stigma towards people who use drugs (*β* = −0.08, *p* < 0.001). See Table 2 for the detailed analyses.

The five moral foundation variables were entered in the third block and were found to explain an additional 21% (*p* < 0.001) of the variance in predicting support for SCS over and above the previously entered variables. Among these variables, greater scores on the *Harm/Care* (β = 0.26, *p* < 0.001) and *Fairness/Reciprocity* (β = 0.17, *p* < 0.001) subscales were associated with more significant support for SCS; higher scores on the *Authority/Respect* (β = −0.10, *p* < 0.01) and *Purity/Sanctity* (β = −0.35, *p* < 0.001) subscales predicted less support. Examination of the squared semi-partial correlations revealed the *Purity/Sanctity* (sr^2^ = 0.06), *Harm/Care* (sr^2^ = 0.03), and *Fairness/Reciprocity* (sr^2^ = 0.01) subscales made the most considerable unique contributions to predicting support for SCS in general. By comparison, *Authority/Respect* and *Ingroup/Loyalty* scores uniquely accounted for a much smaller proportion of the variance (sr^2^ = 0.004 & sr^2^ = 0.0001, respectively).

### Province of residence and municipality size

Results from multiple linear regression analysis revealed significant differences between provinces related to the *Purity/Sanctity* and *Fairness/Reciprocity* subscales in both the adjusted and unadjusted models. Compared to participants from Manitoba, individuals from Alberta and Saskatchewan were found to have lower scores on the fairness/reciprocity scale and higher scores on the purity/sanctity scale. See Table 3 for the complete overview of these analyses. Similarly, participants from medium-sized municipalities (15,000–49,999 people) were found to have significantly higher scores on the *Purity/Sanctity* subscale compared to people from large municipalities (≥ 50,000 people) in the adjusted model. Differences between the small (< 15,000 people) and large municipalities did not reach statistical significance.

**Table 3:** Multiple linear regressions predicting moral foundation scores from municipality size, province, stigma of addiction, and exposure to PWUD

	Unadjusted models				Adjusted^1^ models			
	β	*b*	SE	*p*	β	*b*	SE	*p*
DV: Harm/care								
*Municipality Size*^*2*^								
Large	Ref	–	–	–	Ref	–	–	–
Medium	−0.03	−0.52	0.35	0.14	−0.03	−0.46	0.35	0.19
Small	−0.03	−0.36	0.25	0.15	−0.04	−0.48	0.26	0.07
*Province*								
Manitoba	Ref	–	–	–	Ref	–	–	–
Alberta	−0.10	−0.97	0.25	< 0.001	−0.09	−0.87	0.25	0.01
Saskatchewan	−0.09	−0.09	0.25	< 0.001	−0.09	−0.90	0.25	< 0.001
Stigma of addiction	0.04	0.05	0.03	0.05	0.02	0.02	0.03	0.42
DV: Fairness/reciprocity								
*Municipality Size*								
Large	Ref	–	–	–	Ref	–	–	–
Medium	−0.04	−0.62	0.33	0.06	−0.04	−0.58	0.33	0.08
Small	−0.05	−0.54	0.24	0.02	−0.04	−0.44	0.24	0.07
*Province*								
Manitoba	Ref	–	–	–	Ref	–	–	-
Alberta	−0.12	−1.16	0.23	< 0.001	−0.11	−1.02	0.24	< 0.001
Saskatchewan	−0.11	−1.02	0.24	< 0.001	−0.11	−1.00	0.24	< 0.001
Stigma of addiction	0.03	0.03	0.03	0.29	0.02	0.02	0.03	0.47
DV: Purity/sanctity								
*Municipality Size*								
Large	Ref	–	–	–	Ref	–	–	–
Medium	0.10	2.07	0.47	< 0.001	0.09	1.93	0.48	< 0.001
Small	0.06	0.87	0.34	0.01	0.04	0.63	0.36	0.08
*Province*								
Manitoba	Ref	–	–	–	Ref	–	–	–
Alberta	0.12	1.58	0.33	< 0.001	0.13	1.79	0.34	< 0.001
Saskatchewan	0.15	1.98	0.34	< 0.001	0.15	2.02	0.34	< 0.001
Stigma of addiction	−0.13	−0.22	0.04	< 0.001	−0.13	−0.23	0.04	< 0.001
DV: Authority/respect								
*Municipality Size*								
Large	Ref	–	–	–	Ref	–	–	–
Medium	0.03	0.57	0.37	0.13	0.04	0.68	0.38	0.08
Small	0.005	0.06	0.27	0.83	0.02	0.22	0.28	0.42
*Province*								
Manitoba	Ref	–	–	–	Ref	–	–	–
Alberta	0.07	0.71	0.26	0.007	0.08	0.79	0.27	0.003
Saskatchewan	0.07	0.78	0.27	0.004	0.07	0.70	0.27	0.01
Stigma of addiction	−0.12	−0.16	0.03	< 0.001	−0.11	−0.15	0.03	< 0.001
DV: Loyalty/in-group								
*Municipality Size*								
Large	Ref	–	–	–	Ref	–	–	–
Medium	0.04	0.78	0.38	0.04	0.05	0.90	0.39	0.02
Small	−0.03	−0.34	0.28	0.22	−0.01	−0.14	0.29	0.64
*Province*								
Manitoba	Ref	–	–	–	Ref	–	–	–
Alberta	0.08	0.94	0.27	0.001	0.10	1.08	0.28	< 0.001
Saskatchewan	0.08	0.95	0.28	0.001	0.08	0.88	0.28	0.002
Stigma of addiction	−0.23	−0.32	0.03	< 0.001	−0.22	−0.31	0.03	< 0.001

^1^Adjusted models also included sex, income, and education as covariates

^2^Municipalities coded as (1) Large = more than 50,000 people; (2) Medium = 15,000–49,999 people; (3) Small = fewer than 15,000 people

SE = standard error for the unstandardized regression coefficient (*b*); Ref = reference category

### Stigma

Having more stigmatizing views towards PWUD, as measured by the *Perceived Stigma of Substance Abuse Scale (PSAS)*, was not found to be significantly associated with scores on the *Harm/Care* subscale in either the adjusted or unadjusted analyses (see Table 3). In contrast, stigma was significantly associated with *Purity/Sanctity* scores in both analyses. However, rather than predicting greater scores on the *Purity/Sanctity* scale, greater stigma towards PWUD predicted lower scores on the *Purity/Sanctity* scale (see Table 3). In both the adjusted and unadjusted analyses, stigma was negatively associated with scores on the *Ingroup/Loyalty* and *Authority/Respect* subscales.

### Stigma and history of drug use

Stigma towards PWUD was not significantly associated with respondents declaring a personal history of using cannabis (*r* = 0.01, *p* = 0.62), illicit drugs (*r* = 0.02, *p* = 0.24) or misusing prescriptions (*r* = -0.02; *p* = 0.44).

### Support for SCS

When respondents were asked, “In general, how supportive are you of supervised consumption sites?” most participants (65%) responded that they support SCS in general. However, when participants were asked whether they would support an SCS near their work, the proportion dropped to 59.2%. Similarly, this proportion dropped to an even lower 53% when participants were asked whether they would support an SCS near their home (regardless of moral foundations).

Results from a mixed effect logistic regression revealed that after controlling for the five moral foundations, participants had 0.13 times the odds (*p* < 0.001) of supporting a supervised consumption site near where they live compared to supporting supervised consumption sites in general. By comparison, after adjusting for the five moral foundations, participants had 0.23 times the odds (*p* < 0.001) of supporting a supervised consumption site near where they work compared to supporting supervised consumption sites in general (See Table 4).

**Table 4:** Mixed-effect logistic regression predicting likelihood of support for supervised consumption sites based on the location of the site

	OR	*b*	SE	*p*
*SCS location*				
General support	Ref	–	–	–
Near one’s home	0.10	−2.32	0.16	< 0.001
Near one’s work	0.20	−1.61	0.15	< 0.001

Model also included the following covariates: harm/care, fairness/reciprocity, authority/respect, loyalty/in-group, and purity/sanctity

Abbreviations: SCS, supervised consumption type; SE, standard error for the unstandardized regression coefficient (*b*); *p*, *p*-value; Ref, reference category

### Predicting support for an SCS near one’s home and work

Demographic variables explained 6% (*p* < 0.001) of the variance in predicting support for an SCS near one’s home and one’s work. Individuals with a household income of $100,000 or more annually were found to be less supportive of having an SCS near where they live compared to those making less than $100,000 (refer to Table 5 for the individual comparisons). Similarly, income was also found to predict support for an SCS near one’s work. More specifically, individuals with household incomes of more than $100,000 annually were less supportive relative to those making less than $70,000 annually. In contrast, higher levels of education positively predicted support for having an SCS near one’s home (β = 0.13, *p* < 0.001) and work (β = 0.13, *p* < 0.001). Province of residence was also significantly predictive of support for an SCS near one’s home and work. Similar to the previous models, respondents in Alberta (home: β = −0.17, *p* < 0.001; work: β = −0.16, *p* < 0.001) and Saskatchewan (home: β = -0.11, *p* < 0.001; work: −0.11, *p* < 0.001) indicated that they would be less supportive than Manitobans of having an SCS located near their home or their work. However, the differences in support for SCS between participants from Manitoba and Saskatchewan were not significant in either of the final models (i.e., block 3 of Tables 5 and 6). Municipality size was also found to predict support for an SCS near one’s home and work. After the addition of the moral foundation variables on the third block, however, it was no longer found to significantly predict support for having an SCS near one’s home (see block 3 in Tables 5 and 6).

**Table 5:** Hierarchical regression predicting support for a supervised consumption site near the participants’ home

	Block 1				Block 2				Block 3			
	β	*b*	SE	*p*	β	*b*	SE	*p*	β	*b*	SE	*p*
Age	−0.05	−0.005	0.002	0.03	−0.02	−0.002	0.002	0.31	−0.02	−0.002	0.002	0.49
Sex	< 0.001	< 0.001	0.08	0.99	0.02	0.06	0.08	0.44	−0.03	−0.11	0.08	0.14
Education	0.13	0.18	0.03	< 0.001	0.15	0.20	0.03	< 0.001	0.09	0.12	0.03	< 0.001
*Province*												
Manitoba	Ref	–	–	–	Ref	–	–	–	Ref	–	–	–
Alberta	−0.17	−0.64	0.10	< 0.001	−0.15	−0.58	0.10	< 0.001	−0.09	−0.33	0.09	< 0.001
Saskatchewan	−0.11	−0.42	0.10	< 0.001	−0.11	−0.41	0.10	< 0.001	−0.03	−0.11	0.09	0.21
*Municipality size*												
Large municipality	Ref	–	–	–	Ref	–	–	–	Ref	–	–	–
Medium municipality	−0.07	−0.41	0.14	0.003	−0.07	−0.43	0.14	0.002	−0.03	−0.17	0.12	0.18
Small Municipality	−0.06	−0.24	0.10	0.02	−0.06	−0.26	0.10	0.01	−0.03	−0.13	0.09	0.15
*Annual Income*												
> $100,000	Ref	–	–	–	Ref	–	–	–	Ref	–	–	–
$70,000–$99,999	0.06	0.29	0.11	0.01	0.07	0.31	0.11	0.004	0.06	0.28	0.10	0.005
$30,000–$69,999	0.10	0.40	0.10	< 0.001	0.11	0.43	0.10	< 0.001	0.10	0.41	0.09	< 0.001
$0—$29,999	0.13	0.70	0.13	< 0.001	0.12	0.65	0.13	< 0.001	0.10	0.50	0.12	< 0.001
Stigma of addiction					−0.07	−0.04	0.01	0.001	−0.12	−0.06	0.01	< 0.001
Exposure to PWUD					0.09	0.07	0.02	< 0.001	0.04	0.03	0.02	0.07
Hx of illicit drug use					−0.01	−0.05	0.11	0.68	−0.02	−0.08	0.10	0.43
Hx of cannabis use					0.08	0.31	0.09	< 0.001	0.02	0.05	0.08	0.52
Hx of rx misuse					0.03	0.18	0.14	0.19	0.02	0.12	0.13	0.34
Harm/care									0.17	0.07	0.01	< 0.001
Fairness/reciprocity									0.21	0.09	0.01	< 0.001
Authority/respect									−0.10	−0.04	0.01	0.001
Loyalty/in-group									0.04	0.01	0.01	0.22
Purity/sanctity									−0.28	−0.08	0.01	< 0.001
R^2^ (adjusted R^2^)	0.06 (0.06)				0.09 (0.08)				0.24 (0.23)			

For the sex variable males were coded as 1 and females as 2. Large municipality = greater than 50,000 people; Medium municipality = 15,000–49,999 people; Small municipality = less than 15,000 people

Abbreviations: SE, standard error for the unstandardized regression coefficient (*b*); *p*, *p*-value; Ref, reference category; PWUD, people who use drugs; Hx, history; rx, prescription

**Table 6:** Hierarchical regression predicting support for a supervised consumption site near the participants’ work

	Block 1				Block 2				Block 3			
	β	*b*	SE	*p*	β	*b*	SE	*p*	β	*b*	SE	*p*
Age	−0.05	−0.005	0.002	0.02	−0.04	−0.004	0.002	0.12	−0.03	−0.003	0.002	0.18
Sex	−0.01	−0.04	0.08	0.66	0.01	0.02	0.08	0.77	−0.05	−0.17	0.08	0.03
Education	0.13	0.18	0.03	< 0.001	0.15	0.21	0.03	< 0.001	0.09	0.12	0.03	< 0.001
*Province*												
Manitoba	Ref	–	–	–	Ref	–	–	–	Ref	–	–	–
Alberta	−0.16	−0.61	0.10	< 0.001	−0.15	−0.56	0.10	< 0.001	−0.08	−0.29	0.09	0.001
Saskatchewan	−0.11	−0.42	0.10	< 0.001	−0.11	−0.40	0.10	< 0.001	−0.02	−0.08	0.09	0.37
Municipality size												
Large municipality	Ref	–	–	–	Ref	–	–	–	Ref	–	–	–
Medium municipality	−0.09	−0.56	0.14	< 0.001	−0.10	−0.58	0.14	< 0.001	−0.05	−0.31	0.13	0.01
Small Municipality	−0.09	−0.41	0.10	< 0.001	−0.10	−0.44	0.10	< 0.001	−0.07	−0.30	0.09	0.001
*Annual Income*												
> $100,000	Ref	–	–	–	Ref	–	–	–	Ref	–	–	–
$70,000–$99,999	0.04	0.20	0.11	0.07	0.05	0.23	0.11	0.04	0.04	0.20	0.10	0.05
$30,000–$69,999	0.08	0.33	0.10	0.001	0.09	0.37	0.10	< 0.001	0.09	0.35	0.09	< 0.001
$0—$29,999	0.06	0.31	0.13	0.02	0.05	0.29	0.11	0.03	0.03	0.14	0.12	0.26
Stigma of addiction					−0.09	−0.04	0.01	< 0.001	−0.14	−0.06	0.01	< 0.001
Exposure to PWUD					0.06	0.05	0.02	0.01	0.02	0.01	0.02	0.52
Hx of illicit drug use					−0.01	−0.04	0.12	0.72	−0.02	−0.08	0.10	0.42
Hx of cannabis use					0.11	0.40	0.09	< 0.001	0.04	0.13	0.08	0.12
Hx of rx misuse					0.001	0.01	0.14	0.95	−0.01	−0.05	0.13	0.68
Harm/care									0.19	0.07	0.01	< 0.001
Fairness/reciprocity									0.22	0.09	0.01	< 0.001
Authority/respect									−0.07	−0.03	0.01	0.02
Loyalty/in-group									0.005	0.002	0.10	0.88
Purity/sanctity									−0.29	−0.08	0.01	< 0.001
R^2^ (adjusted R^2^)	0.06 (0.06)				0.09 (0.08)				0.25 (0.24)			

For the sex variable males were coded as 1 and females as 2. Large municipality = greater than 50,000 people; Medium municipality = 15,000–49,999 people; Small municipality = less than 15,000 people

SE, standard error for the unstandardized regression coefficient (*b*); *p*, *p*-value; Ref, reference category; PWUD, people who use drugs; Hx, history; rx, prescription

The second block of variables (i.e., stigma, exposure to PWUD, and a personal history of drug use) was found to explain an additional 3% of the variance over and above the effect of the demographic variables in predicting support for an SCS near one’s home and 2% of the variance in predicting support for SCS near one’s work. Among these variables, exposure to PWUD (β = 0.09, *p* < 0.001) and a personal history of cannabis use (β = 0.08, *p* < 0.001) were positively associated with support for having an SCS located near one’s home. In contrast, stigmatizing views of PWUD were negatively associated with support for having an SCS located near one’s home (β = −0.07, *p* = 0.001). However, after the addition of the moral foundation variables in the third block, exposure to PWUD and a history of cannabis use no longer significantly predicted support for a hypothetical SCS being located near one’s home. Similarly, support for an SCS near one’s work was significantly predicted by a history of cannabis use in block 2, but not block 3; whereas, greater stigma towards PWUD (β = -−0.09, *p* < 0.001) was a significant predictor in both blocks 2 and 3.

The five moral foundation variables explained an additional 15% (*p* < 0.001) of the variance in predicting support for having an SCS near one’s home and 16% (*p* < 0.001) of the variance in predicting support for an SCS near one’s work. Similar to the model predicting support for SCS in general, higher scores on the *Harm/Care* (home: β = 0.17*, p* < 0.001; work: β = 0.19*, p* < 0.001) and *Fairness/Reciprocity* (home: β = 0.21, *p* < 0.001; work: β = 0.22, *p* < 0.001) subscales were associated with significantly more support for having an SCS located near one’s home or work. In contrast, greater scores on the *Purity/Sanctity* (home: β = −0.28,* p* < 0.001; work: β = −0.29,* p* < 0.001) scale were associated with less support for having an SCS near one’s home or work. Greater scores on the *Authority/Respect* scale were also found to predict less support for having an SCS near one’s home (β = −0.10,* p* = 0.001), but not near one’s work (β = −0.07,* p* = 0.02).

Of the five moral foundation variables, the *Purity/Sanctity* (home: sr^2^ = 0.03; work: sr^2^ = 0.04), *Fairness/Reciprocity* (home: sr^2^ = 0.02; work: sr^2^ = 0.02), and *Harm/Care* (home: sr^2^ = 0.01; work: sr^2^ = 0.02) scales were found to make the most considerable unique contributions to predicting a participant’s degree of support for a hypothetical SCS near their home or work. Please refer to Tables 5 and 6 for the individual comparisons.

## Discussion

This cross-sectional observational study explored public attitudes towards SCS using the moral foundations theory and scales regarding stigmatizing views and proximity to PWUD. In terms of socio-demographics, the study found that higher education levels predicted general support for SCS, a finding also reported by some [10, 42, 43] but not supported by others [28].

Analysis using the moral foundations scale revealed a few patterns. Greater scores on the *Harm/Care* and *Fairness/Reciprocity* subscales were associated with more significant support for SCS, while higher scores on the *Authority/Respect* and *Purity/Sanctity* subscales predicted less support. *Purity/Sanctity*, *Harm/Care*, and *Fairness/Reciprocity* subscales contributed to predicting support for SCS, with *Authority/Respect* and *Ingroup/Loyalty* scores accounting for a much smaller proportion. Similarly, Christie et al. [7] found that views of needle exchange programs were positively influenced by the values of *Care* and *Fairness* and negatively influenced by concerns about *Purity* and to a much lesser extent, *Authority.* Violation of *Purity* results in the emotion of disgust [21] and moral outrage, which strongly predict opposition to harm reduction strategies that have come to characterize conservative views. Thus, in addressing conservatives and others who oppose SCS for PWUD, it may be beneficial to address the moral foundations that mean the most to them. For example, public messaging about harm reduction measures that help people stay healthy, hygienic, and safe may land more positively than providing information on the number of lives that may be saved. Alternately, since those who identify as politically conservative tend to prioritize reducing the number of people who use substances rather than reducing the harms of substance use [32]—an aspiration not necessarily at odds with harm reduction—there are many opportunities for Canadians to work on a different aspect of the overdose crisis simultaneously.

Contrary to our hypothesis, having higher scores on *Perceived Stigma of Substance Abuse Scale* [29–31] was not found to be significantly associated with scores on the *Harm/Care* subscale. Stigma was, however, found to be significantly associated with *Purity/Sanctity* scores. Counterintuitively, greater stigma towards PWUD was associated with lower *Purity/Sanctity* scores. Also, contrary to expectations, support for SCS was not predicted by stigma towards PWUD, exposure to people who use drugs, a personal history of using illicit drugs, or a history of misusing prescriptions. However, once the moral foundation variables were added, more significant support for SCS was predicted by less stigma towards PWUD. Also, contrary to our expectations, stigma towards PWUD was not significantly associated with respondents declaring a personal history of using cannabis or illicit drugs or misusing prescriptions. These findings differ from the study by Wild et al. [43], who found an inverse association between personal familiarity with PWUD and stigmatized attitudes toward this outgroup. They also observed a significant positive association between personal familiarity with PWUD and support for harm reduction.

Our study also explored general support for SCS and how that support may change based on the proximity of the SCS near one’s home or place of employment. While general support for SCS was 65% in favour, that number fell when SCS was proposed near the participants’ workplace and fell further when it was proposed to be near the participants’ home. We also found that individuals with higher incomes were less supportive of having an SCS near their home than those with lower incomes. Level of education positively predicted support for having an SCS near one’s home. Stigma, exposure to PWUD, and a history of cannabis use were all associated with support for having an SCS located near one’s home. Higher levels of stigmatizing views towards PWUD were associated with lower levels of support for an SCS near one’s home. It is not surprising that the level of support for SCS decreases from a higher level of theoretical support to lower levels of actual support when located *near my work* and *near my home.* This decrease may be because of concerns that SCS will result in a greater concentration of PWUD in the host community, with a subsequent increase in drug dealing, crime, and public disorder [27]. Neighbours may be concerned about reduced property value, aesthetic deterioration, and decreased safety in the host community. Furthermore, there may be a fear of the stigma of PWUD being projected onto the neighbourhoods that PWUD visits, thereby altering how these areas are perceived [46].

Participants in Alberta and Saskatchewan were less supportive of SCS than participants in Manitoba; the former also indicated that they would be less supportive than those in Manitoba of having an SCS near their home. This was interesting because at the time of the study, Alberta had four brick-and-mortar SCS in three cities, Saskatchewan had one SCS, and Manitoba did not have an SCS. It is unclear whether participants’ experiences with local SCS (in Alberta and Saskatchewan) have impacted their views or if the overall moral and political tenor of the province is such that anti-SCS views arise organically and are unrelated to existing SCS, or whether media reports have fuelled negative attitudes. These findings indicate the complexity of attitudes towards SCS and other complex social issues and the need for further study.

While there is much we do not know about how to have better conversations with people who think differently about SCS, our findings suggest there is room for movement towards the other ideological shore using the language of values and speaking to moral foundations. In my (EP) observation, a common strategy of SCS proponents and advocates is to deliver fact-based information about the number of overdoses reversed or referrals made within an SCS. However, these statistics rarely lead to changed minds [39, 44], particularly for those who are staunchly opposed. Instead, harm reduction advocates may find it helpful to speak to the values of those who oppose SCS. And rather than trying to persuade those who opposed SCS during community consultations, advocates might use a values approach and explore what is important to service users, advocates and community members and work to imbue service provision with these shared values.

A notable proportion of the Canadian public expresses ambivalence [40], opposition (OACP Substance Abuse Committee, 2012) or anger, contempt, and disgust [2, 38] towards SCS. These strong reactions lead to polarization and breakdown of public discourse and disruptions of civility, and ultimately, a lack of services for vulnerable people [22, 40, 43]. While implementing an SCS, public messaging is often focused on providing scientific evidence about the benefits of SCS for PWUD [43]. However, education often fails to overcome public opposition and is not correlated to increasing levels of acceptance [44]. By speaking directly to underpinning moral foundations that undergird strongly held positions that may be otherwise resistant to curiosity, greater progress can be made in productive discourse and advocacy for services in this time of crisis.

## Limitations

This study had several limitations. First, this study utilized a cross-sectional design which does not permit causal claims. Second, data from three provincial survey panels was used and adjusted and weighted to represent provincial demographics; however, there may be nuances to these views that would differ if the population were to be directly surveyed. Third, it is possible that respondents who participated in this study may have had little or no knowledge or experience with SCS so that the survey may have been more hypothetical for some than for others. This may account for the observed differences in attitudes towards SCS among the three provinces. And fourth, while respondents were sampled from three large online panels, it was up to the recipient whether they would participate, which may have introduced bias. Future research that investigates the relationship between actual experience of SCS and attitudes towards SCS is warranted.

## Conclusion

Our research elucidates the values underpinning support for SCS, thus providing a new way to approach the impassioned disagreements this topic tends to engender in public discourse. Therefore, the knowledge generated through this research is essential for public health messaging in addressing the need for SCS in Canada. This study contributes novel and rich quantitative evidence on general public attitudes toward SCS and how those attitudes are shaped by deeply held values called moral foundations. Our findings can inform researchers, policymakers, and health leaders in developing strategies to bring the public on board and increase the acceptance of SCS in their communities.

## Data Availability

No datasets were generated or analysed during the current study.

## Abbreviations

MFT: Moral foundations theory
PWUD: People who use drugs
SCS: Supervised consumption services
